# The Efficacy and Safety of Ureteric Stent Removal with Strings versus No Strings: Which Is Better?

**DOI:** 10.1155/2020/4081409

**Published:** 2020-10-15

**Authors:** Zhenkai Luo, Binbin Jiao, Hang Zhao, Tao Huang, Lin Geng, Guan Zhang

**Affiliations:** ^1^Peking University China-Japan Friendship School of Clinical Medicine, Beijing, China; ^2^Department of Urology, China-Japan Friendship Hospital, Beijing, China; ^3^Graduate School of Peking Union Medical College and Chinese Academy of Medical Sciences, Beijing, China

## Abstract

**Objective:**

To evaluate the current evidence on the effectiveness and safety of ureteric stent removal using strings compared to conventional methods.

**Materials and Methods:**

The electronic databases PubMed, Embase, China National Knowledge Infrastructure (CNKI), and the Cochrane Library were systematically searched up to March 2020. Two reviewers searched the literature, independently extracted the data, and evaluated the quality of the studies according to the inclusion and exclusion criteria. The data analysis was performed with the software program Review Manager 5.3.

**Results:**

Eleven studies with a total of 1809 patients were included in the analysis based on the inclusion criteria. Our meta-analysis showed that visual analogue scale (VAS) scores were significantly lower in the string group than in the conventional group (weighted mean difference (WMD) -2.63; 95% confidence interval (CI) -3.68, -1.58; *P* < 0.00001). In terms of stent dwell time, the string group had an advantage (WMD -9.53; 95% CI -14.20, -4.86; *P* < 0.0001). In addition, no significant differences in the occurrence of urinary tract infection (UTI) (odds ratio (OR) 1.03; 95% CI 0.62, 1.72; *P* = 0.92), emergency room visits (OR 0.99; 95% CI 0.59, 1.67; *P* = 0.97), or other complications (*P* > 0.05) were observed between the two groups.

**Conclusion:**

Our findings suggest that an extraction string is an effective and safe method for the removal of ureteric stents. This method gives patients the benefits of reduced pain and shortened stent dwell time without increasing the risk of UTI. Nevertheless, these findings should be further confirmed through large-volume, well-designed prospective randomized controlled trials (RCTs).

## 1. Introduction

With the advancement of science and technology, the management of urological diseases has gradually become less invasive; ureteric stents have played an important role in this improvement. Ureteric stents are mainly used for relief of ureteral obstruction, treatment of ureteral fistula, and posttreatment of ureteral intervention, among other purposes [1]. Currently, with the wide use of ureteroscopy to remove upper urinary tract stones and detect diseases, ureteric stent placement has become a routine. According to reports, 80% of urologists will place ureteral stents after uncomplicated transurethral lithotripsy [2]. Placement of ureteric stents can facilitate the flow of urine to promote residual stone discharge and decrease the risk of ureteral stenosis [3, 4]. However, ureteral stents remain an optional recommendation after ureteroscopic lithotripsy (URS) according to the American Urological Association (AUA) guidelines [5], and some issues still need to be considered when placing ureteric stents. While ureteral stents are in place, patients feel urgency and discomfort, and there are some related complications, such as infections and encrustations, that negatively impact the quality of life [6, 7]. In addition, the removal of a ureteral stent is usually performed by cystoscopy, and patients not only bear the high cost of surgery but also suffer from pain during the process [8, 9].

In order to solve this problem, some stents with extraction strings affixed to the distal end are applied in the clinic. This design allows patients to pull out the ureteral stent themselves, which leads to a decrease in the retention time as well as morbidity and relative healthcare costs [10]. However, some potential drawbacks, such as urinary symptoms related to extraction, as well as increased risks of urinary tract infection (UTI) and dislodgment, have attracted the attention of urologists [11]. Recently, a study has been performed to compare the two ways of removing ureteral stents [12]. The results are still controversial. However, the studies were few in number and had small sample sizes, which may have caused some bias in the results. Therefore, we conducted this systematic review and meta-analysis of the available literature to evaluate the efficacy and safety of ureteric stent removal using strings. We hope this work will provide a reference for urologists and patients to select the optimal management method.

## 2. Materials and Methods

### 2.1. Search Strategy

We conducted a systematic comprehensive literature search of PubMed, Embase, the China National Knowledge Infrastructure (CNKI), and the Cochrane Library up to March 2020. The keywords “stents”, “ureteric stent”, “string”, “renal stone”, and “cystoscopy” were used to search for articles. These search terms were used individually and in combination. There were no language restrictions on the search process. Additionally, we manually searched the references and citation lists of all relevant reviews. To select studies, we applied a search strategy that conformed to the Preferred Reporting Items for Systematic Reviews and Meta-Analysis (PRISMA) guidelines.

### 2.2. Inclusion and Exclusion Criteria

The following inclusion criteria were used: (1) the study type was a randomized controlled trial (RCT) or a case-control trials (CCTs); (2) the study evaluated the efficacy and safety of ureteric stent removal using strings compared to cystoscopy; (3) the participants were adults with indwelling ureteric stents; (4) no statistically significant difference was found in the basic characteristics of the participants; and (5) at least one of the following outcomes was reported: visual analogue scale (VAS) scores, urinary tract infection (UTI), and postoperative complications.

Studies were excluded if they fulfilled any of the following criteria: (1) the inclusion criteria were not met; (2) the publication was of an incomplete type, such as a conference abstract, letter, comment, or review; (3) ureteric stents were removed by means other than extraction strings or cystoscopy; (4) patients had renal abnormalities (horseshoe kidney or solitary kidney) or existing UTIs.

### 2.3. Data Extraction and Quality Assessment

The selection of literature was performed based on the inclusion and exclusion criteria. Two reviewers (Z.L. and B.J.) independently extracted the data and appraised both quality and content. The data extracted for the analysis included first author, year of publication, country, study design, intervention, sample size, VAS, UTI occurrence, stent dwell time, emergency room visits, and overall complications. Any disagreements were resolved through discussions among all the authors.

We rated the level of evidence (LE) for every included study according to the Oxford Centre for Evidence-Based Medicine Criteria [13]. For the methodological quality assessment, we used the Jadad scale [14] to assess the quality of RCTs and the Newcastle-Ottawa Scale (NOS) [15] to evaluate the quality of CCTs.

### 2.4. Statistical Analysis

The software program Review Manager 5.3 was used to perform all statistical analyses. The weighted mean difference (WMD) and odds ratio (OR) were used to compare continuous and dichotomous variables. All results were reported with 95% confidence intervals (CIs). The *χ*^2^ and *I*^2^ tests (*I*^2^ > 50% was regarded as substantial heterogeneity) were used to assess the heterogeneity of the study data. The fixed-effects models were selected for the meta-analyses if heterogeneity was considered to be low. Otherwise, a random-effects model was used to reduce the effect of statistical heterogeneity. The pooled effects were determined by the *Z* test, and a *P* value < 0.05 was considered statistically significant. Forest plots are used to express the results of the meta-analysis.

## 3. Results

### 3.1. Characteristics of the Selected Studies

**Figure 1:**
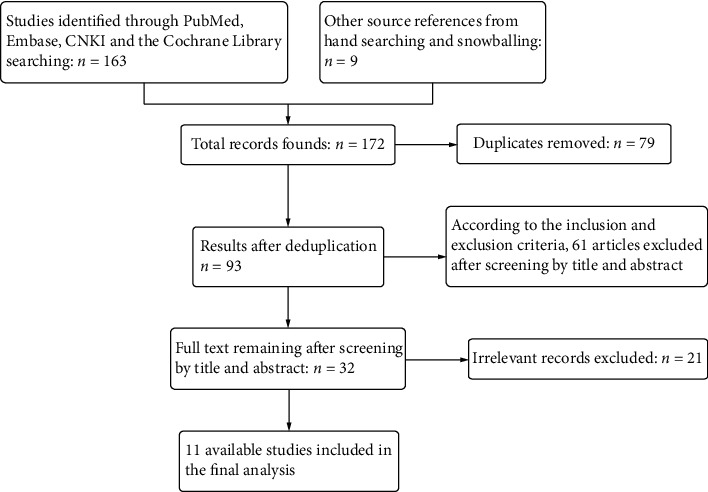
PRISMA flowchart.

172 studies were included according to the search strategy. After screening the abstract and full text, we ultimately included eleven studies [8, 10, 11, 16–23]. The literature selection process is presented in a flowchart (Figure 1). The selected studies included 7 RCTs and 4 CCTs, with a total of 717 cases of strings and 1092 cases of cystoscopy in this meta-analysis. The baseline characteristics and quality assessment of the included studies are summarized in a table (Table 1).

### 3.2. Pain Visual Analogue Scale

**Figure 2:**
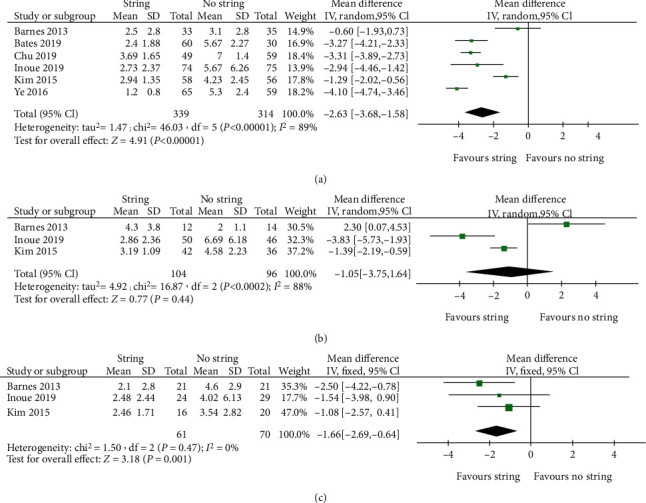
Forest plots and meta-analyses. (a) Overall VAS. (b) Male VAS. (c) Female VAS. 95% CI: 95% confidence intervals; df: degrees of freedom; Fixed: fixed-effects model; Random: random-effects model; IV: inverse variance; SD: standard deviation.

Six studies provided data on VAS scores. A random-effects model was used due to the high heterogeneity (*I*^2^ = 89%). The combined results showed that the string group had a lower score than the cystoscopy group (WMD -2.63; 95% CI -3.68, -1.58; *P* < 0.00001) (Figure 2(a)). In addition, we performed a subgroup analysis depending on gender. The analysis results showed no significant difference in males (WMD -1.05; 95% CI -3.75, 1.64; *P* = 0.44) (Figure 2(b)), but the female group showed a difference in favour of extraction with strings (WMD -1.66; 95% CI -2.69, -0.64; *P* = 0.01) (Figure 2(c)).

### 3.3. Stent Dwell Time

**Figure 3:**
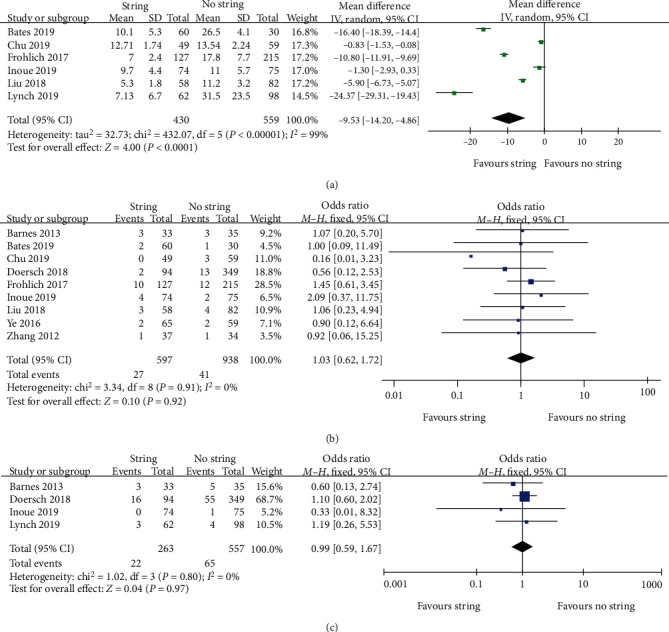
Forest plots and meta-analyses. (a) Stent dwell time. (b) UTI. (c) ER visit. 95% CI: 95% confidence intervals; df: degrees of freedom; Fixed: fixed-effects model; Random: random-effects model; IV: inverse variance; SD: standard deviation.

Referring to the stent dwell time, a total of six studies that included 989 participants met the inclusion criteria. The pooled result by the random-effects model (*I*^2^ = 99%) demonstrated that the string group incurred a shorter indwelling time than the cystoscopy group (WMD -9.53; 95% CI -14.20, -4.86; *P* < 0.0001) (Figure 3(a)).

### 3.4. UTI

The outcome of UTI was reported in nine studies, with a total of 68 events in 1535 participants. With no heterogeneity (*I*^2^ = 0), a fixed-effects model was selected. The results showed that the differences between the two groups were not statistically significant (OR 1.03; 95% CI 0.62, 1.72; *P* = 0.92) (Figure 3(b)).

### 3.5. Emergency Room Visit

With respect to the incurrence of emergency room (ER) visits, five studies were included in this meta-analysis. Based on the results of a fixed-effects model (*I*^2^ = 0), no significant difference was found between the string group and cystoscopy group. (OR 0.99; 95% CI 0.59, 1.67; *P* = 0.97) (Figure 3(c)).

### 3.6. Complication

**Figure 4:**
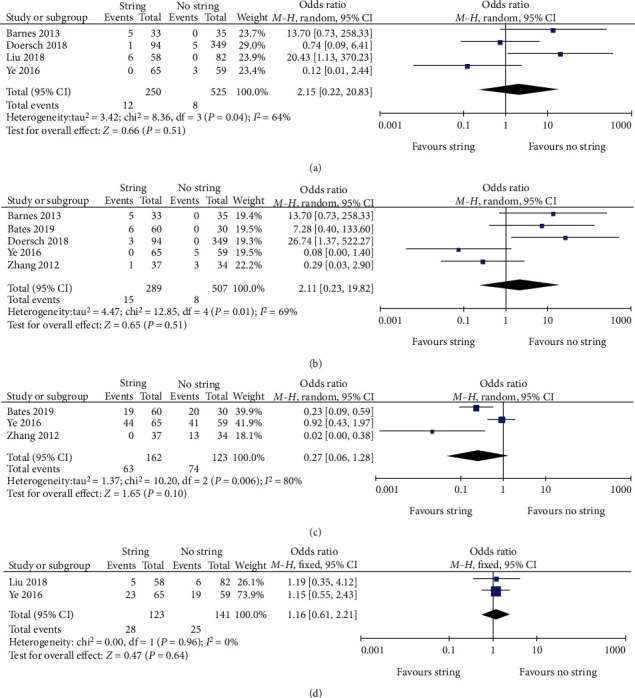
Forest plots and meta-analyses. (a) Stent dislodgement. (b) Early pull. (c) Haematuria. (d) LUTS. 95% CI: 95% confidence intervals; df: degrees of freedom; Fixed: fixed-effects model; Random: random-effects model; IV: inverse variance; SD: standard deviation.

We analysed the occurrence of complications, including stent dislodgement, early pulling, haematuria, and lower urinary tract symptoms (LUTS). The overall results showed no significant difference between the two groups regarding the incidence of these complications (*P* > 0.05). The results are shown in Figure 4.

### 3.7. Sensitivity Analysis and Publication Bias

**Figure 5:**
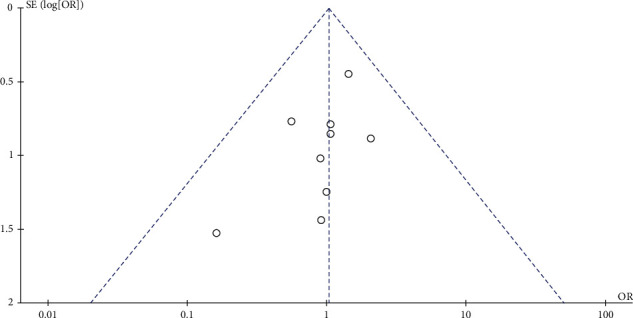
Funnel plot of UTI for publication bias.

To decrease the effect of high heterogeneity, we performed a sensitivity analysis by subgroup of RCTs; the results are presented in Table 2. Except that the stent dwell time was significantly different, other results showed no change in significance compared with the original analysis, indicating that the results of our meta-analysis were stable. We also conducted funnel plots to detect publication bias in this meta-analysis. With no apparent asymmetry, the results indicated no obvious publication bias (Figure 5).

## 4. Discussion

For every urologist, ureteric stents are a commonly used internal drainage device; however, the ureteric stent does not have a long history. Zimskind et al. first performed ureteral catheterization under cystoscopy in 1967 to relieve urinary tract obstruction [24]. Due to the limitations of conditions at that time, the catheters were easily displaced and prolapsed, which was not generally accepted. Since the introduction of the double J tube into the clinic by Finney [25] in 1978, the research and application of the ureteric stent has made great progress in the field of urology. With the function of internal drainage and ureter support, its application has been affirmed clinically. At present, the most commonly used clinical material of ureteric stents is polyurethane material, which can be left in the body for 6 months [26]. However, as foreign bodies, the complications of indwelling catheters gradually increase with dwell time. The impact of quality of life (QoL) on patients during tube placement is also obvious. The distal end of the ureteric stent continuously stimulates the triangle of the bladder, especially during the micturition and prestorage period, showing urinary sensation, urgency, and dysuria. It has been reported that after double J tube insertion, the QoL of 80% of patients is affected, 58% of patients reduce work intensity due to discomfort of tube placement, and nearly half of patients require medical intervention to relieve tube-related symptoms [27]. Therefore, without affecting the effect of the ureteric stent, shortening the retention time as much as possible is the best choice for patients. Unfortunately, in most cases, the urologist decides the extraction date, and patients need to travel to the hospital to undergo the removal procedure by cystoscopy. The fear of pain during the removal process or complex appointment circuit may affect the patient's extraction of the ureteric stent on time, which extended the indwelling time. In contrast to the conventional extraction method, with extraction strings attached to the bladder end of the ureteric stent placed in the urethral opening, patients and patients can remove the stent by themselves. The efficacy and safety of these two methods have gradually gained clinical attention.

As mentioned above, pain during the extraction process is a challenge for patients. Our meta-analysis shows that compared to the conventional method, the extraction string will bring less pain for patients. The outcome is consistent with previously published literature [28]. In most cases, cystoscopy is used to remove the ureteric stent as a conventional method, but for patients with difficulty in extubation, a ureteroscope is also selected. However, these factors both increase patient pain and the risk of urethral mucosa injury [28]. In contrast, with extraction strings, patients can control the strength to pull out the stent slowly, which relieves pain and reduces the stimulation of the system, especially for elderly patients. From our included study, only Barnes et al. (2013) reported no difference in pain between the two extraction methods [8]. We found that they used intraurethral lidocaine jelly for stent removal, which may decrease pain associated with stent extraction. Apart from this, the pain in conventional methods may be associated with the clinical experience of the surgeon, the type of cystoscopy, and the use of any adjunctive medications or local anaesthesia [29–31]. However, unlike females, men have a longer urethra. Even when using local anaesthetic drugs, most patients can still feel severe pain caused by cystoscopy insertion [32]. Our results also demonstrated that the difference in feeling pain between males and females and the pain score among males was still higher than that among females using strings. Therefore, for male patients, some local anaesthetic drugs may be used when extracting strings to relieve pain. Only three studies reported that the VAS depended on the subgroup gender, with different process details, which may cause bias to outcome. We hope that more studies with detailed pain scores depend on different criteria, such as gender, location, and time, to obtain more reliable outcomes.

The stent dwell time is also a key impact on patient QoL. A longer stent dwell time was reported as a risk factor for more stent-related symptoms [33]. For patients after retrograde intrarenal surgery, a longer indwelling time of ureteric time will not contribute to a higher stone-free rate but will increase the incidence of relative complications. At present, there is still controversy regarding the retention time of stents. Consequently, a ureteric stent should be left with a short time needed. However, the left time of the stent depends on the surgeon's experience, and other nonmedical factors determined include the clinical schedule. Therefore, the stent dwell time may be longer than individual plans [10]. Patients in the string group can remove the stent at home to ensure a suitable time, without time wasting in appointment and travelling to the hospital. The results of this meta-analysis also confirm this, and we found that the overall stent dwell time was shorter in patients with stents removed via extraction strings. In our study, the relative detail data were not shown in some included studies, such as lack of standard deviation or represented in graphical, thus could not be added to the meta-analysis. In addition, they set language restrictions and reduced the inclusion of available literature, which also contributed to the difference in conclusions. Due to the CCTs included, determination of stent dwell time will require more high-quality RCTs.

Regarding the greatest concern of urologists, our pooled data indicated no difference in the incidence rate of UTI between the string group and conventional group. There is no definite evidence that string placement will increase the rate of UTI. Some doctors may feel that the string will conduct bacteria into the body and increase the rate of postoperative UTI and bacteriuria, especially for female patients due to their relatively short urethra [34]. In our included study, no significant difference in UTI was reported. In addition, compared to the invasive conventional method, extraction string outside the urethra is a noninvasive operation that brings less risk of interfering with the internal environment of the body and causing infection of exogenous pathogenic microorganisms [35]. Due to the use of antibiotics and the emphasis on infection, the rate of UTI is not obvious in the two extubation methods, except those patients with very long indwelling times. Therefore, we recommend that urologists consider placing the ureteric stent with extraction strings without concerning the risk of UTI.

During stent placement, some patients may go to the emergency room for help due to possible unexpected events, such as severe pain and stent breakage. We summarized it as an ER visit and found no difference in the two methods regarding the incidence of ER visits. According to all studies, we conclude that stent dislodgement, early pull, haematuria, and LUTS are complications to analysis. The results showed that there were no significant differences between the groups. It is worth mentioning that for stent dislodgement, a high incidence rate (15.1%) in the string group was reported [8]. Furthermore, Althaus et al. [36] showed that women with a stent string experienced stent dislodgment compared with males (24.4% vs. 5.3%; *P* < 0.05). The higher dislodgment rate in women may be associated with a relatively short urethra or inadvertent tugging on the extraction string when bathing or after voiding. However, in a study by Inoue et al., they reported that no stent dislodgment appeared in either group [11]. They mentioned that they explained the details and the necessity of the stent string to the patients and tied a new knot to prevent accidental touch. Therefore, we assume that stent dislodgement is related to informing in detail and patient self-management. To better decrease the rate of stent dislodgement, a urologist should place a shorter extraction string outside the urethra and firmly fix it accordingly. At the same time, informing the importance and caution of extraction strings to patients is also indispensable. Due to the limited number of studies and the relatively small sample size to observe the relative complications, more studies are required to verify the safety of the strings in ureteric stent removal.

According to a review of the literature, ureteral stents cause a variety of urinary tract symptoms, stent-related pain, and additional problems [7, 37]. During stent placement, patients' physical and psychosocial health will be affected and have a negative impact on functional capacity and work performance. To better evaluate the influence of ureteric stents, Joshi et al. [27] described the ureteral stent symptom questionnaire (USSQ). Due to the complexity of USSQ, only two of the included studies [8, 19] reported the relative outcome. The results show that the general domain scores on the USSQ were not different between the two groups. We hope the USSQ could be simplified to apply more conveniently, and USSQ is recommended in related studies to make the results more comparable in the future.

Because the use of related medical equipment and drugs is avoided, the cost of stent extraction string is very low. As different currencies are used from studies in different countries, we could not perform a meta-analysis to assess the cost. However, according to the description in some studies, it is easy to see the obvious difference in cost. Liu et al. [20] reported that the cost of ureteral stent removal for patients with extraction strings was lower (8.97 ± 3.07 vs. 455 ± 0 CNY; *P* < 0.05). In a study by Lynch et al., their department saved €23,790 during the 7-month study period due to the successful removal of 61 stents with extraction strings [12, 21]. For this advantage, several studies have identified the use of a string for self-removal of stents after URS as a cost-saving measure [12]. Despite this, due to cultural differences in different regions, surgeon and patient attitudes towards the use of stent extraction strings are different based on countries. According to Loh-Doyle et al.'s study [31], the most common use of extraction strings is in Canada (25.6%), followed by the United States (12.6%). For patients, they often mind the stent removal method. Barnes et al.'s study mentioned 202 potential candidates who refused to participate, as they did not want to remove their stents themselves [8]. Indeed, in China, most urologists choose the cystoscope to remove the stents, and patients are pleased to this. Therefore, it is not easy to say which methods are best, and the most suitable choice should depend on the patient's own condition and human factors.

There are several limitations in our meta-analysis. First, some included RCTs failed to describe the blinding methods and detailed randomization concealment, which may cause conclusion bias. Although sensitivity analysis showed that the results were relatively stable, potential bias by the included CCTs was inevitable. In addition, heterogeneity for some outcomes among studies was found to be high, including VAS, stent dwell time, and some complications. The high heterogeneity can be explained by the difference in surgical experience, postoperative management, and outcome definitions and measurements. Finally, the difference in the stent model and the aim of placing the stent (urolithiasis or hydronephrosis) may lead to bias. The limitations identified should be taken into consideration when interpreting these results. We hope that more large-volume and high-quality RCTs will be designed to validate our findings.

## 5. Conclusion

This meta-analysis indicates that an extraction string is an effective and safe method for the removal of ureteric stents. Compared with the conventional method, removal with a string is associated with reduced pain and shortened stent dwell time with no increase in the risk of UTI. There were no significant differences in other complications, such as stent dislodgement, haematuria, or LUTS. Although patients may benefit from these advantages, the use of extraction strings still needs to be based on clinical decisions and patient willingness.

## Figures and Tables

**Table 1:** Summary of comparative studies included in meta-analysis.

Study	Country	Study design	Intervention		Sample size		LE	Study quality
			Trial	Control	Trial	Control		
Barnes et al. 2014 [8]	USA	RCT	String	Cystoscopy	33	35	2a	4^∗^
Bates et al. 2019 [16]	UK	CCT	String	Cystoscopy	60	30	2b	7^#^
Chu et al. 2019 [17]	China	RCT	String	Cystoscopy	49	59	2a	3^∗^
Doersch et al. 2018 [18]	USA	CCT	String	Cystoscopy	94	349	2b	8^#^
Fröhlich et al. 2017 [10]	Switzerland	CCT	String	Cystoscopy	127	215	2b	7^#^
Inoue et al. 2019 [11]	Japan	RCT	String	Cystoscopy	74	75	2a	3^∗^
Kim et al. 2015 [19]	Korea	RCT	String	Cystoscopy	58	56	2a	3^∗^
Liu et al. 2018 [20]	China	RCT	String	Cystoscopy	58	82	2a	3^∗^
Lynch et al. 2020 [21]	Ireland	CCT	String	Cystoscopy	62	98	2b	8^#^
Ye et al. 2016 [22]	China	RCT	String	Cystoscopy	65	59	2a	3^∗^
Zhang et al. 2012 [23]	China	RCT	String	Cystoscopy	37	34	2a	3^∗^

RCT: randomized controlled trial; CCTs: case-control trials; LE: level of evidence. ^∗^Using the Jadad scale (score from 0 to 5). ^#^Using the Newcastle-Ottawa Scale (score from 0 to 9).

**Table 2:** Sensitivity analysis results.

Outcomes	No. of studies	Sample size		Heterogeneity (total)				MD or RR (95% CI)	*P* value (total)
		Trail	Control	Chi^2^	df	*I* ^2^%	*P* value		
Overall VAS	5	279	284	45.44	4	91	<0.00001	-2.49 [-3.75, -1.24]	*P* = 0.0001
Stent dwell time	3	181	216	82.66	2	98	<0.00001	-2.70 [-6.34, 0.95]	*P* = 0.15
UTI	6	316	344	2.12	5	0	0.83	0.97 [0.47, 1.98]	*P* = 0.92
Emergency room visit	2	107	110	0.13	1	0	0.72	0.57 [0.16, 1.95]	*P* = 0.37
Stent dislodgement	3	156	176	6.78	2	70	0.03	3.08 [0.14, 66.12]	*P* = 0.47
Early pull	3	135	128	6.44	2	69	0.04	0.64 [0.04, 9.86]	*P* = 0.75

CI: confidence interval; MD: mean difference; RR: risk ratio.

## Data Availability

The datasets used and/or analysed during the current study are available from the corresponding author on reasonable request.
